# Growing together: Longitudinal trajectory of posttraumatic growth among suicide-loss survivors and its interpersonal predictors

**DOI:** 10.1192/j.eurpsy.2023.2378

**Published:** 2023-07-19

**Authors:** Y. Levi-Belz

**Affiliations:** 1 Ruppin Academic Cener, Emek hefer, Israel

## Abstract

**Introduction: Background:**

Recent studies have indicated that grieving after suicide loss can be particularly complex and traumatic. However, studies have recognized the opportunity for personal growth among suicide-loss survivors.

**Objectives:**

This study signifies an effort to develop a comprehensive understanding of the underlying interpersonal facilitators of posttraumatic growth (PTG) among suicide-loss survivors in a longitudinal design.

**Methods:**

Participants included 189 suicide-loss survivors (155 females), aged 21–73, who completed questionnaires of thwarted belongingness (TB), perceived burdensomeness (PB), and self-disclosure at T1. Moreover, participants were assessed on PTG levels at T1, 18 months (T2), and 42 months (T3).

**Results:**

The integrated mediation model indicated that both TB and PB contributed to the PTG trajectory. PB and self-disclosure contributed to PTG at T3 beyond the PTG trajectory across time. We also found self-disclosure to mediate the association of TB and PTG at T2 and T3.

**Conclusions:**

These findings suggest that interpersonal factors play critical roles in contributing to PTG over time among suicide-loss survivors. Basic psychoeducational interventions designed to foster interpersonal behaviors may facilitate achieving PTG among survivors in the aftermath of suicide loss.

**Disclosure of Interest:**

Y. Levi-Belz Shareolder of: no, Grant / Research support from: no, Consultant of: no, Employee of: no, Paid Instructor of: no, Speakers bureau of: no

